# Torsion chronique d'une rate baladeuse chez un adolescent: à propos d'un cas

**DOI:** 10.11604/pamj.2016.24.15.7554

**Published:** 2016-05-05

**Authors:** Hamidou Dème, Léra Géraud Akpo, Seynabou Fall, Nfally Badji, Ibrahima Ka, Mohamadou Lamine Guèye, Mouhamed Hamine Touré, El Hadj Niang

**Affiliations:** 1 Service de Radiologie et Imagerie Médicale, CHU Aristide Le Dantec, Dakar, Sénégal; 2 Service de Médecine Interne, CHU Aristide Le Dantec, Dakar, Sénégal; 3 Service de Chirurgie Générale, CHU Aristide Le Dantec, Dakar, Sénégal

**Keywords:** Rate baladeuse, torsion pédiculaire, tomodensitométrie abdominale, échographie, splénectomie, Wandering spleen, torsion of the pedicle, CT scan, ultrasound, splenectomy

## Abstract

La rate baladeuse ou errante est une anomalie rare, qui est le plus souvent décrite chez l'enfant. Ses complications parmi lesquelles figure la torsion de son pédicule sont fréquentes et peuvent engager le pronostic vital du patient. Nous rapportons un cas de torsion chronique du pédicule d'une rate baladeuse chez un patient de 17 ans, au long passé de douleurs épigastriques. Le tableau clinique était marqué par une masse épigastrique spontanément douloureuse, évoluant depuis 48 heures. L’échographie abdominale objectivait une rate ectopique hétérogène, hypertrophiée, en position épigastrique et un hématome sous capsulaire. Au doppler, on notait une torsion du pédicule splénique à deux tours de spires et un petit flux sur l'artère splénique. La tomodensitométrie abdominale avec injection de produit de contraste montrait un défaut de rehaussement parenchymateux d'une grosse rate ectopique épigastrique et un hématome sous capsulaire. Le diagnostic de torsion chronique du pédicule d'une rate baladeuse, compliquée de nécrose et d'hématome sous capsulaire était retenu. Il a été réalisé une splénectomie. Les suites opératoires étaient simples. Nous discutons l'apport de l’échographie et de la TDM dans le diagnostic de la torsion chronique du pédicule d'une rate baladeuse.

## Abstract (en)

Wandering or migrating spleen is a rare anomaly which is usually described in children. Complications, which include pedicle torsion, are common and can be life-threatening. We report the case of a 17 year-old patient with a long past medical history of epigastric pain suffering from wandering spleen with chronic torsion of the pedicle. The clinical picture was marked by spontaneously painful epigastric mass, evolved over the past 48 hours. Abdominal ultrasound objectified heterogeneous hypertrophied ectopic spleen in epigastric position and a subcapsular hematoma. Doppler showed a torsion of splenic pedicle which was untwisted 2 turns and a small blood stream on the splenic artery. Abdominal CT scan with contrast injection showed a lack of parenchymal enhancement of large epigastric ectopic spleen and a subcapsular hematoma. The diagnosis of wandering spleen with chronic torsion of the pedicle complicated by necrosis and subcapsular hematoma was confirmed. The patient underwent splenectomy. The postoperative course was uneventful. We here discuss the contribution of ultrasound and CT scan in the diagnosis of wandering spleen with chronic torsion of the pedicle.

## Introduction

La rate baladeuse ou errante est une anomalie rare qui est le plus souvent retrouvée chez l'enfant [1]. C'est une mobilité anormale de la rate, due à une hyperlaxité ou à une agénésie de son ligament suspenseur d'origine congénitale ou acquise [2, 3]. Sa présentation clinique est déroutante car pouvant siéger dans tous les quadrants de l'abdomen. Elle est souvent découverte au stade de complications, notamment lors de la torsion de son pédicule. Nous rapportons un cas de torsion chronique d'une rate baladeuse compliquée d'un infarctus splénique chez un adolescent en insistant sur l'apport de l'imagerie.

## Patient et observation

**Figure 1:**
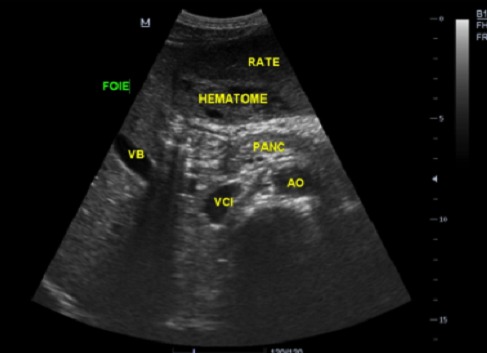
Coupe échographique transversale sous xiphoïdienne montrant une rate hétérogène en situation épigastrique

**Figure 2:**
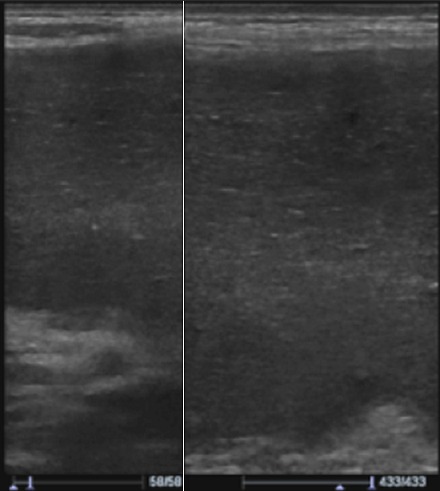
Coupe transversale avec une sonde linéaire de haute fréquence montrant l’échostructure hétérogène de la rate

**Figure 3:**
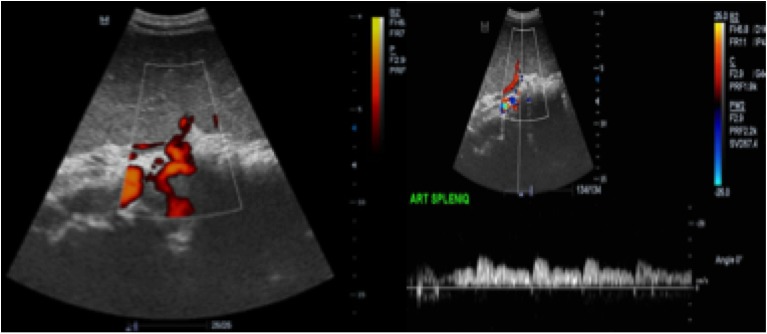
A) doppler énergie montrant 2 tours de spires sur la l'artère splénique; B) doppler pulsé, petit flux sur l'artère splénique

**Figure 4:**
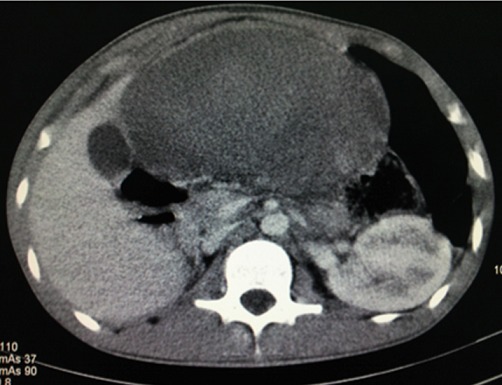
Coupe axiale de TDM abdominale après injection de PDC, montrant un parenchyme splénique hétérogène non rehaussée, un discret rehaussement de la capsule, un hématome sous capsulaire et une absence de rehaussement des vaisseaux spléniques

**Figure 5:**
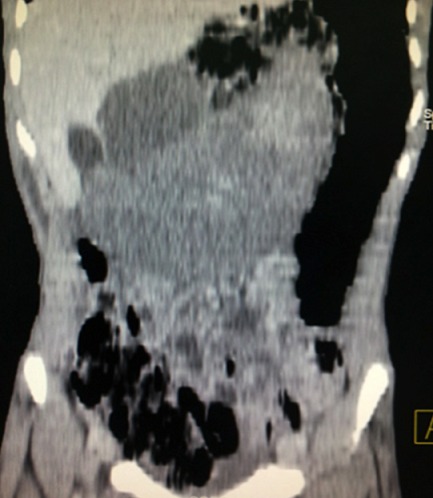
Reconstruction coronale montrant la rate hétérogène épigastrique

Un adolescent de 17 ans consultait pour des douleurs épigastriques aiguës, fixes, intenses et permanentes, évoluant depuis 48 heures, sans vomissements ni troubles du transit. Dans ses antécédents, on notait des douleurs épigastriques modérées, intermittentes évoluant depuis 6 mois, d'allure non ulcéreuse. A l'examen, la conscience était claire, la température était de 37°6 C, le pouls était de 90 BPM, la tension artérielle de 110 / 75mmHg et la fréquence respiratoire de 22 cycles/min. On notait une masse épigastrique douloureuse, ferme, à surface lisse dont les limites étaient difficilement appréciables. Le reste de l'abdomen était souple et il n'y avait pas d'ascite. L'hémogramme avait trouvé une anémie hypochrome normocytaire (taux d'hémoglobine =9,6g/dl); une thrombocytose (taux de plaquettes= 1.388.000/mm3); il n'y avait pas d'hyperleucocytose. Le reste du bilan biologique notamment: la C réactive protéine, le taux de prothrombine, *l'international normalized ratio* (INR), le temps de céphaline activée (TCA), la fibrinémie, l'ionogramme sanguin et la lipasémie était normal. L'imagerie était réalisée avec un échographe modèle Mindray^®^ DC7 et une tomodensitométrie (TDM) 2 barrettes, modèle Siemens^®^. L’échographie montrait une vacuité de la loge sous phrénique gauche avec une rate en position ectopique épigastrique, augmentée de taille, hétérogène et un hématome sous capsulaire (Figure 1, Figure 2). Le doppler énergie montrait un aspect de torsion du pédicule splénique à 2 tours de spires avec la persistance d'un petit flux artériel au doppler pulsé (Figure 3). La TDM confirmait la vacuité de la loge splénique avec une rate ectopique épigastrique hypodense hétérogène avec une plage de densité liquidienne 20 Unités Hounsfield (UH) sous capsulaire. Après injection de produit de contraste iodé, il n’était pas noté de rehaussement du parenchyme splénique; mais un discret rehaussement de la capsule (Figure 4 et Figure 5). Par ailleurs, les vaisseaux spléniques n’étaient pas objectivement opacifiés et on notait une malrotation et une ptose rénale droite. Une exploration chirurgicale permettait de confirmer la torsion du pédicule splénique à 2 tours de spires dans le sens anti-horaire, associée à une rate d'allure nécrotique. Il a été réalisé une splénectomie. Les suites opératoires étaient simples.

## Discussion

La rate baladeuse est une entité rare à ne pas méconnaître. Sa prévalence est faible (0,2%), et elle est surtout l'apanage de l'enfant [1]. La prédominance féminine est rapportée dans la population adulte [4, 5]. Elle est secondaire à une hyperlaxité ou à une agénésie des ligaments suspenseurs de la rate, relevant d'une anomalie congénitale ou acquise [6]. La forme congénitale est due à un défaut d'accolement du mésogastre postérieur avec allongement du pédicule splénique, ce qui confère à la rate une certaine liberté dans la cavité abdominale. Quant à la forme acquise, plusieurs facteurs ont été incriminés, notamment: la distension gastrique, la splénomégalie, le traumatisme abdominal et la grossesse [6, 7]. Le plus souvent, la rate baladeuse est asymptomatique et sa découverte est fortuite lors d'un examen abdominal ou d'un examen d'imagerie (incidentallome). Elle peut également se manifester par des douleurs abdominales intermittentes témoignant de crises de torsion et de détorsion spontanées, comme cela était le cas chez notre patient [6]. Lorsqu'un volvulus chronique de rate s'installe, le pédicule splénique est enroulé, l'artère splénique est rétrécie et la veine splénique est comprimée ou obstruée [8]. Souvent, le patient développe une splénomégalie, un hypersplénisme, un infarctus splénique avec des adhérences périspléniques, à l'origine d'une douleur abdominale d'abord aiguë puis chronique comme cela était le cas chez notre patient [9, 10]. La torsion de la rate baladeuse favorisée par sa mobilité, son poids et la longueur de son pédicule peut être irréversible et se manifester par un abdomen aigu chirurgical où la douleur abdominale vient au premier plan, associée parfois à des nausées et des vomissements et à de la fièvre. La palpation abdominale recherche des signes péritonéaux et une masse abdominale ou pelvienne [7]. L'association de ce tableau d'abdomen aigu à une masse abdominale mobile doit faire évoquer le diagnostic de torsion de rate baladeuse et faire indiquer une imagerie pour confirmer le diagnostic. L’échographie abdominale constitue l'examen d'imagerie de première intention [5]. Elle permet d'affirmer le diagnostic de torsion de la rate baladeuse devant la vacuité de la loge splénique et la mise en évidence d'une masse abdominale rappelant l’échostructure splénique comme cela était le cas chez notre patient. En outre, l'absence de vascularisation au Doppler est un argument en faveur de la torsion [7, 11]. L’écho doppler visualise la diminution ou l'absence de flux dans la veine et l'artère splénique [12]. Chez notre patient un petit flux était présent sur l'artère splénique.

Cependant l’échographie connaît des limites en rapport avec un iléus, une obésité, une hypertrophie du lobe gauche du foie pouvant être confondue avec la rate. Dans ces cas, le diagnostic de torsion peut être difficile et le recours à la TDM est nécessaire [5]. En effet, la TDM est la modalité d'imagerie de choix pour le diagnostic de rate baladeuse, surtout lorsqu'une torsion du pédicule est suspectée ou si l’échographie n'est pas contributive [5]. Elle confirme le diagnostic en montrant une loge splénique vide avec une masse abdominale ou abdomino-pelvienne rappelant l'aspect de la rate et ne prenant pas le produit de contraste après injection intraveineuse. Le pédicule tordu est visualisé à la TDM sous la forme d'un tourbillon ou *« whirl sign »*représentant les tours de spire sur le pédicule splénique [5, 7]. L'infarctus splénique, stade ultime de la torsion, est suspecté à la TDM devant le *« rim sign »* qui correspond à une hyperdensité de la capsule splénique contrastant avec l'hypodensité du parenchyme en rapport avec la formation d'une circulation collatérale [5]. Chez notre patient, la TDM a confirmé la vacuité de la loge splénique et le défaut de rehaussement du parenchyme splénique et un discret rehaussement de la capsule périphérique. Une fois que le diagnostic de cette complication est posé, l'exploration chirurgicale s'impose. Elle confirme le diagnostic et permet d'adapter le traitement. En effet, en l'absence de nécrose splénique, la détorsion peut être réalisée avec une splénoplexie permettant de fixer la rate dans sa position anatomique normale. En cas de nécrose splénique, la splénectomie est indiquée [5]. Chez notre patient, il a été réalisé une splénectomie, vu l'infarcissement de la rate.

## Conclusion

Le syndrome de la rate baladeuse est une affection rare, surtout décrite chez l'enfant. La torsion chronique de son pédicule en est une complication grave pouvant mener à un infarctus splénique. Le diagnostic doit être évoqué devant une douleur abdominale aiguë associée à une masse abdominale mobile. L’écho doppler et la TDM, permettent un diagnostic précoce, gage d'un traitement conservateur.
